# Choroidal macrovascular and capillary alterations in eyes with idiopathic epiretinal membranes

**DOI:** 10.5935/0004-2749.2022-0369

**Published:** 2024-03-05

**Authors:** Ahmet M. Hondur, Yavuz Kemal Aribas

**Affiliations:** 1 Department of Ophthalmology, Gazi University Faculty of Medicine, Ankara, Turkey; 2 Department of Ophthalmology, Hacettepe University Faculty of Medicine, Ankara, Turkey

**Keywords:** Epiretinal membrane/surgery, Vitrectomy, Choroid/ pathology, Choroid/blood supply, Tomography, optical coherence/methods, Optical coherence tomography angiography, Humans

## Abstract

**Purpose:**

To evaluate the choroidal vascular alterations and effect of surgical treatment in the setting of idiopathic epiretinal membranes.

**Methods:**

The structure of the choroid was studied in 33 patients with unilateral idiopathic epiretinal membrane using optical coherence tomography with enhanced depth imaging and optical coherence tomography angiography. Eyes with epiretinal membrane underwent 25-gauge vitrectomy with epiretinal membrane and internal limiting membrane peeling. The choroidal vascularity index, Haller layer/choroidal thickness ratio, and choriocapillaris flow density were used to evaluate changes in choroidal structure after surgery and compare with the healthy fellow eyes.

**Results:**

The choroidal vascularity index and Haller layer/choroidal thickness ratio of the eyes with epiretinal membrane were higher than those of the fellow eyes at baseline (p=0.009 and p=0.04, respectively) and decreased postoperatively compared with preoperative values (p=0.009 and p=0.001, respectively). The choriocapillaris flow of eyes with epiretinal membrane was lower than that of the fellow eyes at baseline (p=0.001) and increased after surgery compared with the preoperative value (p=0.04). The choroidal vascularity index, Haller layer/choroidal thickness ratio, and choriocapillaris flow values of the healthy fellow eyes were comparable at baseline and final visit. In eyes with epiretinal membrane, the final choroidal vascularity index correlated with the final choriocapillaris flow (r=-0.749, p=0.008) in the multivariate analysis.

**Conclusion:**

Idiopathic epiretinal membrane appears to affect the choroidal structure with increased choroidal vascularity index and Haller layer/ choroidal thickness ratio and decreased choriocapillaris flow. These macrovascular (choroidal vascularity index and Haller layer/choroidal thickness) and microvascular (choriocapillaris flow) alterations appear to be relieved by surgical treatment of the epiretinal membranes.

## INTRODUCTION

The idiopathic epiretinal membrane (ERM) is a common vitreoretinal interface disorder characterized by a fibrocellular proliferation on the inner retinal surface. It can lead to vertical and tangential traction and may cause structural changes in the retina.Eyes with macular involvement and visual disturbances such as decreased visual acuity or metamorphopsia can benefit from vitrectomy and ERM peeling^(1)^, and internal limiting membrane peeling can be added to prevent recurrences^(2)^.

The choroid contains blood vessels surrounded by an interstitial stromal tissue and nourishes the other retina. The foveola, which is the central part of the retina that is providing the best visual acuity, is devoid of inner retinal layers and is exclusively supplied by the choroid. A precise study of the structural features of the choroid has become possible with the development of new imaging technologies. Particularly, enhanced depth imaging (EDI) with optical coherence tomography (OCT) and choriocapillaris flow density (CF) assessment with the OCT angiography (OCT-A) have been widely adopted to segment and analyze the choroid. Detailed analysis of the choroid may augment our understanding of its functional status. Thus, the choroidal vascularity index (CVI) has been utilized to examine the choroidal vascular changes in various ocular and systemic diseases^(3,4,5,6)^.

A few reports of ERM-related choroidal changes and its surgical treatment have utilized different methods and have not yielded fully consistent results^(6,7)^. In this study, we aimed to evaluate concomitant changes in the CVI, Haller layer/choroidal thickness (H/C) ratio, CF, and subfoveal choroidal thickness in idiopathic ERM with comparison to healthy fellow eyes.

## METHODS

The study followed the tenets of the Declaration of Helsinki, and the study protocol was approved by the Institutional Review Board of Gazi University [approval no. E133167, December 11, 2020]. This retrospective cohort study included 33 consecutive patients with unilateral idiopathic grade 3 or 4 ERM who underwent surgery. ERM grading was conducted as previously described^(8)^. The healthy fellow eyes of these patients were used as controls.

All patients underwent a comprehensive ophthalmologic examination at baseline and after surgery, including measurement of the best-corrected visual acuity (BCVA) with the Snellen chart, axial length measurements using optical biometry, anterior-segment examination, intraocular pressure measurement with the pneumotonometer, and fundus examination. EDI-OCT scans were obtained with the Spectralis OCT (Heidelberg Engineering, Heidelberg, Germany), and OCT-A images were recorded with the Angiovue Imaging System (RTVue XR AVANTI, Optovue Inc., Fremont, CA, USA).

The exclusion criteria were as follows: axial myopia (>26 mm axial length) and advanced hyperopia (>5 diopters or <21 mm axial length), significant lens opacities (greater than NO3, NC3, C3, or P2 level opacity according to the lens opacity classification scheme)^(9)^, glaucoma, ERM grades 1 and 2^(8)^, other retinal diseases, previous retinal surgery, and history of uveitis or ocular trauma. Patients with incomplete follow-up and poorquality OCT and OCT-A images (signal strength index <60 on a 100-point scale) were also excluded. The mean follow-up duration was 12.2 ± 3.1 months, and the postoperative OCT and OCT-A scans at 6 ± 1 months were used for comparison.

### Surgical procedure

Under local anesthesia, all ERM eyes underwent 25-gauge pars plana vitrectomy with ERM and ILM peeling after staining with trypan blue (Tekno Epi Blue, TEKNOMEK, Turkey). The surgeries for ERM were performed by a single, experienced vitreoretinal surgeon. The retinal periphery was checked for retinal tears/holes with scleral indentation, and a fluid-air exchange was performed at the end of the surgery. Air sulfur-hexaflouride (SF_6_) (Tecnogases SF_6_, TEKNOMEK, Turkey) exchange was performed in a single eye with an incidental retinal tear.

### Calculation of the CVI

Images were recorded using the EDI mode of the Spectralis OCT (Heidelberg Engineering), which is a scanning diode laser of 840 ± 10 nm. After mydriasis, the patient’s head and chin were properly positioned, and an inverted image was acquired from the retina while the patient maintained fixation on the internal fixation light. The image was automatically reversed so that the chorioretinal interface would be adjacent to the zero delay. Thirteen sections and 768 A-scans that are composed of 100 averaged scans were captured in a rectangle including the macula and optic nerve. The intersection point of the vertical and horizontal scans was checked manually before image acquisition.

**Figure 1:**
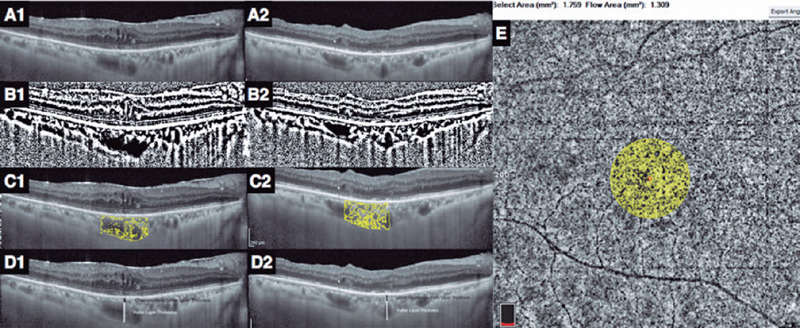
Enhanced depth imaging (EDI) optical coherence tomography (OCT) scan passing through the foveal center of each eye was selected at baseline (A1). Postoperatively, the EDI-OCT scan was matched to the one passing through the central foveal segment at baseline (A2). The image was converted to 8 bits, and thresholding was applied (B1, baseline; B2, postoperatively). Each binarized image was then converted to an RGB image, and the luminal area was determined using the color thresholding tool. The total choroidal, luminal, and stromal areas were calculated in the central 1500 µm (C1, baseline; C2, postoperatively). The Haller layer/choroidal thickness ratio was calculated as the ratio of the Haller layer thickness to the total choroidal (Haller layer and Sattler–choriocapillaris complex) thickness (D1, baseline; D2, postoperatively). The choriocapillaris fow was measured by OCT angiography as the ratio of the fow area to the non-fow area in a circle of 750 µm centered at the fovea (E).

In each eye, the horizontal EDI-OCT scan passing through the fovea was selected for image analyses (Figure 1A1). Postoperatively, the EDI-OCT scan was matched to the one passing through the central foveal segment at baseline (Figure 1A2). The central choroidal thickness was determined as the distance between the lower boundary of the retinal pigment epithelium and the choroid-scleral interface^(10)^. The CVI was computed as the ratio of the luminal area to the total choroidal area. The images were converted to 8 bits, and image thresholding adjustment was applied using ImageJ (version 1.47, National Health Institute, Bethesda, MA, USA) to highlight the vascular lumens (Figure 1B1 and B2). The choroidal area in OCT scans was binarized as reported in the literature^(11)^. Then, each binarized image was converted to the RGB format, and luminal areas were highlighted using the color thresholding tool (Figure 1C1 and C2). The total choroidal, luminal, and stromal areas were calculated within the central 1500 µm. Bright pixels were defined as the choroidal interstitial area, whereas dark pixels were defined as the vascular luminal area^(3,11)^.

### Measurement of the Haller layer thickness and H/C ratio

The choroidal vascular layers were segmented manually. Measurements were performed by two graders, and the mean difference between their measurements was 4.4% ± 1.9% (range, 3%-8%). The Haller layer was delineated as the outer zone of choroidal vessels that were >100 µm^(10)^. The H/C ratio was defned as the ratio of the Haller layer to the total choroidal thickness (Figure 1D1 and D2)^(4)^.

### OCT-A measurements

The 6 × 6 mm OCT-A scans with a signal strength index ≥60 and without any motion artifact were used for analysis. OCT segmentation was automatically performed using the integrated module. The vascular densities of the inner and superficial capillary plexuses, CF, and central foveal thickness were measured using an integrated software. The CF was measured as the ratio of the flow area to the non-flow area in a 750 µm-radius circle, which was centered at the fovea (Figure 1 E)^(4)^.

### Statistical analysis

IBM SPSS Statistics for Windows version 22.0 (IBM Corp., Armonk, NY, USA) was used, and the significance level was set to p<0.05. The conformity of continuous variables to normal distribution was evaluated using visual (histogram and probability graphs) and analytical methods (Kolmogorov–Smirnov/Shapiro-Wilk tests). For continuous variables, ERM eyes were compared with their fellow eyes using dependent variables t-test or Wilcoxon signed ranks depending on conformity to normal distribution. For comparison of categorical variables, the chi-square test was used. Relationships were investigated with the Spearman rank correlation test.

## RESULTS

The baseline characteristics of the patients and their eyes by group are shown in table 1. No difference in intraocular pressure, axial length, and ratio of pseudophakic eyes was found between the idiopathic ERM eyes and fellow eyes. The ERM eyes demonstrated a higher CVI and H/C ratio and a lower CF than their healthy fellow eyes. The baseline central choroidal thickness was slightly lower in ERM eyes than in the fellow eyes and tended to shift toward the thickness of the fellow eyes postoperatively; however, these differences and changes were not significant (Tables 1 and 2).

**Table 1:** Baseline characteristics of the patients and their eyes.

Age		68.2 ± 10.0 years	
Sex		Female: 19 (58 %), male 14 (42 %)	
	**ERM Eyes (n:33)**	**Fellow Eyes (n:33)**	**p-value**
Pseudophakia, n (%)*	15 (45 %)	16 (48 %)	0.805
Intraocular Pressure	15.6 ± 2.5 mmHg	15.3 ± 2.5 mmHg	0.732
Axial Length	23.7 ± 1.75 mm	23.94 ± 1.81 mm	0.675
Choroidal and Retinal Parameters			
Central foveal thickness	371.9 ± 90.6 µm	293.9 ± 57.4 µm	**0.009**
Central choroidal thickness	220.7 ± 47.2 µm	249.0 ± 70.9 µm	0.074
Choroidal vascularity index	0.60 ± 0.04	0.58 ± 0.03	**0.009**
Haller/choroid thickness ratio	0.69 ± 0.08	0.64 ± 0.09	**0.04**
Choriocapillaris flow density	0.56 ± 0.18	0.70 ± 0.04	**0.001**

ERM= Epiretinal membrane; n= Number; *= The remaining eyes were phakic, no eye was aphakic.

**Table 2:** Comparison of the postoperative values of ERM eyes with their baseline values and fellow eyes

	ERM Eyes at Baseline	p-value	ERM Eyes Postoperatively	p-value	Fellow Eyes*
BCVA, logMAR	0.45 ± 0.27	**<0.001**	0.23 ± 0.26	**<0.001**	0.02 ± 0.04
Central foveal thickness (µm)	371.9 ± 90.6	0.215	345.4 ± 85.9	0.185	298.2 ± 63.4
Central choroidal thickness (µm)	220.7 ± 47.2	0.055	234.8 ± 39.7	0.217	252.3 ± 65.7
Choroidal vascularity index	0.60 ± 0.04	**0.009**	0.58 ± 0.03	0.761	0.58 ± 0.03
Haller/choroid thickness ratio	0.69 ± 0.08	**0.001**	0.63 ± 0.08	0.827	0.63 ± 0.09
Choriocapillaris flow density	0.56 ± 0.18	**0.04**	0.69 ± 0.06	0.463	0.71 ± 0.06

BCVA= Best Corrected Visual Acuity; ERM= Epiretinal membrane; *= The values of the fellow eyes for comparison were obtained simultaneously with the postoperative values of ERM eyes.

After ERM peeling, the postoperative CVI and H/C ratio of the ERM eyes decreased and the CF increased significantly compared with their baseline values. The postoperative CVI, H/C ratio, and CF of the ERM eyes were not different from those of the fellow eyes (Table 2).

In ERM eyes, significant correlations were found between the final CVI and CF (r=-0.743, p=0.002) and between the change in logMAR visual acuity and the change in CVI (r=0.45, p=0.008). Multivariate analysis revealed that only the correlation between the final CVI and final CF (r=-0.749, p=0.008) was significant.

## DISCUSSION

Indocyanine green angiography has been the gold standard imaging modality for the evaluation of choroidal vascular pathologies that lead to dye leakage, staining, or non-perfusion. However, direct and segmental analyses of the structural characteristics of the choroid such as vascular caliber and choriocapillary alterations have only been possible with OCT and OCT-A, respectively. Particularly, EDI scans of OCT and CF assessment with OCT-A have recently been widely accepted and utilized for studying choroidal vascular changes in various disorders^(3,4,5,7,11,12,13)^.

In ERM, fibrocellular proliferation on the inner retinal surface causes vertical and tangential traction and can lead to distortional changes in the retina^(1)^. In addition to the retinal changes generated by the ERM, our results demonstrate that the traction may be transmitted to the choroid and lead to changes in the macro- and microvascular structure of the choroid. Increased CVI and H/C ratio in EDI scans represent macrovascular changes, whereas diminished CF in OCT-A signifies microvascular alterations in ERM eyes compared with the healthy fellow eyes (Table 1). In addition, surgery appears to reverse these alterations to a comparable level with the unaffected fellow eyes (Table 2).

In the literature, choroidal thickness was the first parameter to be studied related to ERM, and studies have reported contradictory results^(14,15,16,17)^. The present study did not disclose any significant change in the choroidal thickness in ERM. However, the baseline central choroidal thickness in ERM eyes tended to be thinner than that in the healthy fellow eyes and appeared to increase postoperatively. These minor changes in choroidal thickness may imply the initial displacement of the foveal center by the ERM away from the choroidal central point-where the choroid is thickest-and a postoperative return toward its original location. Another consequence of this tractional displacement is that postoperative OCT images do not pass through the exact same section of the choroid (Figure 1A1 and A2) because consecutive OCT images are matched to the retinal landmarks of the baseline OCT image.

As the choroidal thickness is affected by various ocular and systemic factors^(18,19)^, the CVI has been described as a more consistent and stable parameter for the examination of choroidal vasculature and structure than choroidal thickness^(3,11)^. A few studies have examined ERM-related CVI changes. In a combined group of eyes with ERM and macular hole, the CVI decreased after vitrectomy. However, this study did not report any comparison of ERM eyes with their fellow eyes and any results related to choriocapillary changes^(7)^. In another recent study, the perfusion of choriocapillaris, Sattler layer, and Haller layer was evaluated with OCT-A. The baseline values of these parameters were similar in ERM eyes and their fellow eyes. However, after ERM surgery, the choriocapillaris perfusion increased, whereas that of the Sattler layer decreased, and the authors concluded that the resolution of vitreomacular traction might lead to the shift in the blood flow from the Sattler layer to the choriocapillaris. However, they did not note any change in the Haller layer^(20)^.

Although our methodology of studying the big choroidal vessels differed from this recent OCT-A study^(20)^, our results also imply improved CF and less pooling of the blood in the upstream Sattler layer after surgical treatment. However, in contrast to this previous study^(20)^, our analysis of the big choroidal vessels using the well-established methodology of CVI^(3,11)^ and H/C ratio^(4)^ also demonstrated changes in the Haller layer.

Hypotheses for ERM-related choroidal changes have also been proposed. High levels of vascular endothelial growth factor (VEGF) following mechanical stress to the rat retinal pigment epithelium were demonstrated in vitro^(21)^. ERM eyes with age-related macular degeneration or diabetic macular edema are less responsive to anti-VEGF treatment^(22,23)^. Thus, a high VEGF level was proposed to be a probable causative factor in ERM-related choroidal changes^(7)^. However, VEGF prevents choriocapillaris endothelial cell apoptosis and is essential for the integrity of the choriocapillaris endothelium^(24)^. Hence, high VEGF levels would be expected to have a positive effect on the vascularity of choriocapillaris. By contrast, our results imply a diminished CF, which makes the VEGF hypothesis implausible for the ERM-associated choriocapillary changes.

Vitreomacular traction caused by an ERM was proposed to lead to a reduced tissue pressure at the retinal area of traction, which would lead to a higher hydrostatic pressure difference between the retinal tissue and capillary lumen and therefore edema in the affected retina^(14)^. Our results imply that the tractional effect of ERM may extend beyond the retina to the underlying choroid and affect the choriocapillary bed differently. The choriocapillary bed is highly fenestrated; thus, the hydrostatic pressure difference between the choroidal tissue and choriocapillary lumen is much lower. Consequently, choriocapillary bed alteration appears to be capillary distortion and decreased flow. In addition, the upstream choroidal arteries lack the autoregulation capacity of the retinal arterioles and hence cannot respond to decreased choriocapillary blood flow.

The decreased flow at the choriocapillary bed level appears to lead to stagnation of the upstream blood flow and congestion in upstream big arteries of the Haller and Sattler layers. This may be perceived as an increased H/C ratio and CVI in EDI-OCT. In favor of this hypothesis, the resolution of tractional forces after ERM surgery appears to restore the choriocapillaris flow and resolve the upstream congestion in the present study. The moderate negative correlation (r=-0.749, p=0.008) between the final CF and CVI of ERM eyes also support our hypothesis.

Among various other parameters, a weaker correlation was also observed between the change in logMAR visual acuity and the change in CVI (r=0.45, p=0.008); however, multivariate analysis did not confirm this clinical relationship of vascular changes. The multiple complex features of ERM such as the direction of traction, duration, severity, presence of macular edema, and many others may affect visual acuity. In addition, difficulties associated with OCT-A of choriocapillary bed may have precluded the disclosure of such a correlation between CF and visual acuity.

In clinical practice, CVI may not be calculated directly because it involves sophisticated image processing. Therefore, we previously proposed a simpler parameter, which is the H/C ratio^(4)^. This ratio can be easily assessed using the measurement tools of many OCT systems. Therefore, it may be more simply implemented into clinical practice than the CVI. In our practice, it appears to be the most noticeable choroidal change during qualitative evaluation of EDI-OCT images. On the contrary, the CVI has the advantage of incorporating vascularity and changes in the Sattler layer.

The present study demonstrated decreased CF and increased CVI and H/C ratio in eyes with idiopathic ERM. Surgical treatment appeared to reverse these changes to a level similar to the unaffected fellow eyes. These findings imply that the choroidal micro- and macrovascular circulation are affected in idiopathic ERM. The small sample size and retrospective design are among the limitations of this present study. Further studies may help elucidate the precise mechanism of choroidal circulatory alterations and the value of these parameters as a follow-up tool in vitreoretinal interface disorders.
